# Analysis of Maternal and Congenital Syphilis Rates at a New Jersey University Hospital

**DOI:** 10.3390/children11060697

**Published:** 2024-06-06

**Authors:** Paige Heiman, Vineet Bhandari, Sarah Davenport, Krystal Hunter, Melissa Micallef, Alla Kushnir

**Affiliations:** 1 Cooper Medical School of Rowan University, Camden, NJ 08103, USA; heiman98@rowan.edu (P.H.); hunter-krystal@cooperhealth.edu (K.H.); micallef-melissa@cooperhealth.edu (M.M.); kushnir-alla@cooperhealth.edu (A.K.); 2 Division of Neonatology, Department of Pediatrics, Cooper University Hospital, Camden, NJ 08103, USA; 3 Children’s Hospital of Philadelphia, Philadelphia, PA 19104, USA; davenporse@chop.edu; 4 Department of Pediatrics, University of Pennsylvania, Philadelphia, PA 19104, USA; 5 Cooper Research Institute, Cooper University Hospital, Camden, NJ 08103, USA

**Keywords:** congenital syphilis, syphilis, STIs, COVID-19

## Abstract

Syphilis and congenital syphilis (CS) cases have been rising in the U.S. and internationally since the 2000s. Social factors have been shown to increase the risk of CS transmission. The COVID-19 pandemic may have contributed to increased syphilis transmission. We aimed to quantify the rise in congenital syphilis (CS) rates at a large urban hospital and the impact of the COVID-19 pandemic on CS rates. We completed a retrospective chart review of 61 pregnant women with a positive test or previous diagnosis of syphilis at an urban academic hospital between 1 January 2016 and 1 June 2022. Maternal syphilis and CS rates increased over the 5 years (*p* < 0.001), particularly pre- and post-COVID-19 (*p* < 0.001). Of the mothers studied, 34.6% received adequate prenatal care, 62.7% received adequate screening, and 81.3% received adequate treatment. Stillbirth was noted in 6.6% of pregnancies. Of liveborn infants, 97.6% received appropriate treatment, and 45.1% received adequate follow-up. CS development was significantly associated with homelessness (*p* = 0.028) and past opioid use (*p* = 0.031). We concluded that maternal syphilis and CS rates have increased at our hospital, particularly during the COVID-19 pandemic. Access to prenatal care and timely maternal treatment are target areas for improvement.

## 1. Introduction

Syphilis rates have steadily been rising both in the U.S. and around the world since the early 2000s, when the disease had reached a low point at 2.1/100,000 in the United States [1,2,3]. Congenital syphilis (CS) incidence rates have largely mirrored those of syphilis in reproductive-aged women. In 2013, the rate of CS in the U.S. was 9.2 cases per 100,000 live births, representing the first increase in rates since 2008 [4]. In 2022, the national CS rate reached 102.5 cases per 100,000 live births, a 30.6% increase from 2021 and a 937.3% increase from 2013 [5]. CS can cause a variety of perinatal and postnatal complications, including placental abnormalities, preterm birth, hydrops fetalis, central nervous system abnormalities, bony and facial deformities, intellectual disability, and deafness [1,6,7]. In 2022, 1.4% of CS cases resulted in infant death and 6.2% resulted in stillbirth, while 37.8% were born alive with clinical signs of infection [5]. These increased rates may be affected by various social determinants of health, including race, substance use during pregnancy, recent incarceration, and homelessness [6,8]. 

The manifestations of CS in a newborn and its effect on development can be devastating. However, this disease can be easily prevented with proper, timely detection and treatment of syphilis in pregnant women. Studies evaluating the efficacy of CDC-recommended treatment for syphilis in pregnancy have shown near 100% success in preventing CS transmission [9]. In 2018, the CDC identified four areas of missed opportunities for prevention of CS development nationally, which include lack of adequate maternal treatment despite timely diagnosis, lack of timely prenatal care, lack of syphilis testing despite timely prenatal care, and late identification of maternal disease [8]. The COVID-19 pandemic added new difficulties, with all-cause syphilis rates at a large urban hospital increasing from 1.2% to 1.8% between June 2019 and June 2020 [10]. This increase was particularly influenced by an increase among women of reproductive age, who made up 9.3% of cases pre-pandemic compared to 31% during the pandemic [10]. 

Our study sought to quantify trends in syphilis rates among pregnant women and CS rates at a large urban hospital over a 5-year period, which included the onset of the COVID-19 pandemic, to identify points of intervention within testing and treatment protocols that may reduce disease incidence and severity.

## 2. Materials and Methods

This study was a retrospective chart review with data collection from electronic medical records (EMR) utilized at our institution. 

All pregnant women who were seen at our urban tertiary University Hospital and had an ICD-10 or Laboratory Result (LRR) code for positive rapid plasma reagin (RPR), fluorescent-treponemal antibody testing (FTA), or syphilis of any stage, or those who had a syphilis diagnosis included in the problem list in the form of an ICD-10 code from 1 January 2016 to 1 June 2022 were included. Mothers with known sero-fast syphilis post-adequate treatment prior to pregnancy and those without adequate data in the EMR were excluded from the study. The charts of infants resulting from each of these pregnancies were also reviewed in detail.

Data on each pregnancy, such as adequacy of screening, treatment, and prenatal care, were extracted from maternal charts. Charts of infants exposed to syphilis in utero were reviewed for the development of CS and characteristics such as the adequacy of screening and follow-up. Definitions of adequacy were derived from the American Academy of Pediatrics (AAP) Red Book Report 2021 edition [11]. For this study, a positive CS diagnosis was defined as either a proven or possible CS diagnosis. A proven case was defined as one in which the infant has an abnormal physical exam, an abnormal/incomplete evaluation, or an RPR > 4× the maternal RPR. A possible case was defined as one in which the mother received inadequate or no treatment and the infant had a normal exam and positive RPR result that was less than or equal to fourfold maternal RPR. Adequate maternal screening was defined as RPR or venereal diseases research laboratory (VDRL) testing at 28 weeks’ gestation and again in the third trimester or delivery, as defined by the state of NJ [12]. Adequate prenatal care was defined as ≥1 visit per trimester. Appropriate treatment was a penicillin-based regimen based on disease stage. Adequate infant testing was defined as neonatal serum RPR and adequate infant outpatient follow-up was defined as visits at 2, 4, 6, and 12 months, with RPR or VDRL every 2–3 months until it became non-reactive. 

Descriptive data regarding prenatal care, delivery, and both neonatal and maternal demographics (including race, ethnicity, work status, marital status, and insurance type) was also collected. Demographic data, including race and ethnicity, were all acquired from EMR designations and were all self-reported by the patient upon hospital registration. We included race and ethnicity in the present study as significant differences in CS development have been reported in the current literature, and we aimed to further elucidate this relationship. This was reported as White, Black, Hispanic, or Other, which included women of any other racial group or women for whom race was not defined in the EMR. Information regarding maternal co-infection with HIV or Hepatitis C and prior syphilis treatment was also assessed. Relevant social determinants of health were assessed, including maternal alcohol use, smoking status, current or past opioid use, homelessness, and incarceration history. The significance of these factors’ association with CS development relative to a lack of CS development was analyzed.

Rates of maternal and congenital syphilis at our hospital per year were calculated and trends analyzed using the Cochran Armitage Test of Trend. Analyses were conducted using SPSS 27 software (IBM, Armonk, NY, USA). Rates between pre-COVID (2016–2019) and post-COVID (2020–2022) periods were compared using Chi-square analysis. Rates of CS at our hospital were compared to NJ state and national rates per year obtained from the Center for Disease Control using a statistical comparison of slopes. The rate of vertical transmission was calculated as CS incidence/maternal syphilis incidence per year multiplied by 100. A comparison of the prevalence of various social determinants of health between infants who were given a CS diagnosis and those who were not was also carried out using a Chi-square analysis. A *p*-value < 0.05 was considered statistically significant. 

## 3. Results

### 3.1. Demographics

A total of 13,535 women were evaluated between the years of 2016 and 2022, with 61 maternal-infant pairs analyzed after qualifying for testing positive for syphilis and meeting inclusion criteria. Of the 61 infants analyzed, 31 were identified as having a proven or possible CS diagnosis. Maternal and infant demographics are shown in Table 1 and Table 2, respectively. Notably, not all charts had complete data available for evaluation, so percentages represent only those charts with available data. In addition to the four stillbirths in this study, one infant who was classified as a positive CS case died at 8 weeks due to cardiac arrest after they had been discharged from the hospital. The birth defects observed in infants diagnosed with CS in this study included one atrial septal defect, one patent foramen ovale, and one hypospadias. One of the stillbirths was found to have fetal heterotaxy with dextrocardia, a common atrioventricular canal, and asplenia at birth. Stillbirths were not considered positive CS cases as data on their RPR could not be obtained. Additionally, some live infants not diagnosed with CS had birth defects, including one cranial deformity and one dextrocardia. 

### 3.2. Maternal and Infant Data

**Figure 1:**
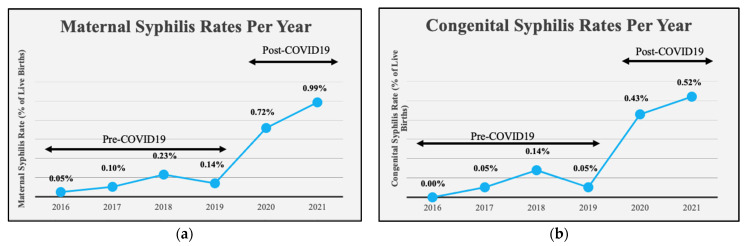
Trends analyzed with Cochran Armitage Test of Trend. Pre- (2016–2019) vs. post-COVID-19 (2020–2021) analyses performed with Chi-Square. (**a**) Rates of maternal syphilis prevalence per year (*p* < 0.001). Exact ratios per year are as follows: 1/2087, 2/2016, 5/2197, 3/2196, 15/2096, 21/2122, respectively. These ratios represented as cases per 100,000 live births, are as follows: 47.9, 99.2, 227.5, 136.6, 715.6, 989.6, respectively. Post-COVID-19 syphilis rates are significantly higher than pre-COVID-19 (*p* < 0.001). (**b**) Rates of CS incidence per year (*p* < 0.001). Exact ratios per year are as follows: 0/2087, 1/2016, 3/2197, 1/2196, 9/2096, 11/2122, respectively. These ratios represented as cases per 100,000 live births are as follows: 0, 49.6, 136.5, 45.5, 429.4, 518.4, respectively. Post-COVID-19 CS rates are significantly higher than pre-COVID-19 (*p* < 0.001).

**Figure 2:**
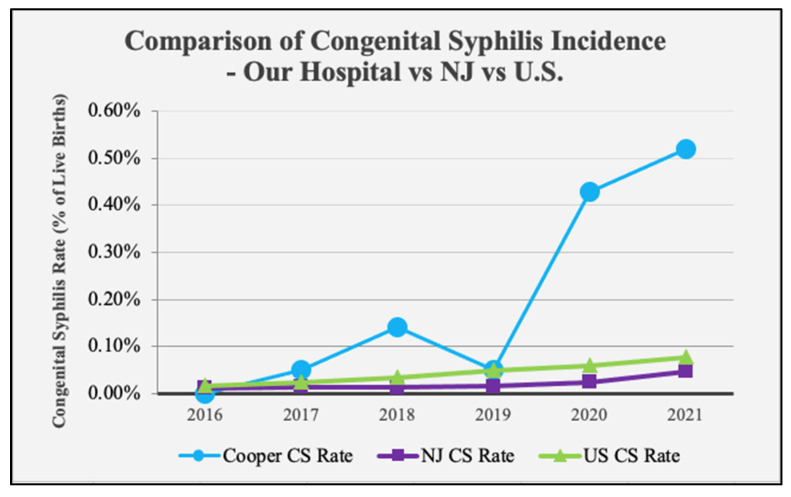
CS incidence at our hospital compared to NJ state and national CS incidence through the end of 2021. Our hospital rates were significantly higher than both state and national rates in 2018 (*p <* 0.001 and *p* = 0.011, respectively), 2020 (*p <* 0.001 for both), and 2021 (*p <* 0.001 for both). NJ and national rates retrieved from published CDC data [13].

**Figure 3:**
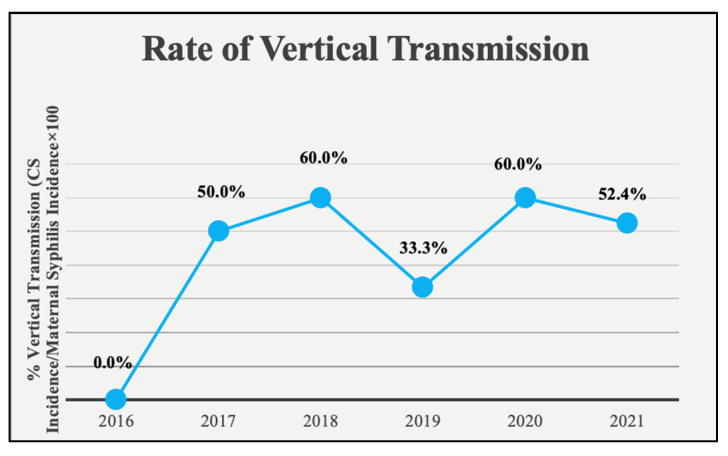
Rate of vertical transmission calculated as CS incidence/maternal syphilis prevalence × 100. Exact ratios per year are 0/1, 1/2, 3/5, 1/3, 9/15, 11/21.

Our results showed that rates of both maternal syphilis and CS have risen at our hospital over the 5-year study period. Syphilis and CS rates per year are shown in Figure 1. Maternal syphilis was calculated as the number of pregnant women who tested positive for syphilis at our hospital/the number of live births at our hospital per year. Exact ratios per year for 2016, 2017, 2018, 2019, 2020, and 2021, are as follows: 1/2087, 2/2016, 5/2197, 3/2196, 15/2096, 21/2122, respectively. These ratios, represented as cases per 100,000 live births, are as follows: 47.9, 99.2, 227.5, 136.6, 715.6, 989.6, respectively. CS was calculated as the number of infants diagnosed with proven or possible CS at our hospital/the number of live births at our hospital per year. The exact ratios for CS rates per year for 2016, 2017, 2018, 2019, 2020, and 2021 are as follows: 0/2087, 1/2016, 3/2197, 1/2196, 9/2096, 11/2122, respectively. These ratios, represented as cases per 100,000 live births, are as follows: 0, 49.6, 136.5, 45.5, 429.4, 518.4, respectively. Syphilis rates in both mothers and infants remained relatively constant in the pre-COVID period (2016–2019) but showed a significant increase at the onset of the COVID-19 pandemic and beyond (*p <* 0.001) (Figure 1). CS rates at our hospital mirrored both NJ and U.S. rates from 2016 to 2018, except for a slight significant increase in 2018 (*p <* 0.001 and *p* = 0.011 for NJ and U.S., respectively). However, in 2020 and 2021 CS rates at our hospital well exceeded state and national rates (*p <* 0.001) (Figure 2). Additionally, we calculated the rate of vertical transmission per year as a measure of our screening and treatment protocols. This value was 0% in 2016, when there were no CS cases at our hospital. Vertical transmission jumped to 50% and 60% in 2017 and 2018, then dropped slightly in 2019 to 33.3%. Vertical transmission rose again in 2020 to 60% before dropping back to 52.4% in 2021. This trend continued in the first 6 months of 2022, where we saw a 42.9% vertical transmission rate (Figure 3). Assessment of screening and treatment success is represented in Table 3.

Maternal social factors, including race, zip code, employment status, substance use, homelessness, and concomitant infections, among others, were compared between infants born with CS and those not born with CS. Infants born to mothers who had a history of opioid use, homelessness or were employed at delivery were found to be significantly more likely to develop CS (*p =* 0.03 for all). These comparisons are shown in Table 4.

## 4. Discussion

It has been well established that syphilis rates have been rising both nationally and internationally. The CDC reported a 24% increase in primary and secondary syphilis rates among reproductive-aged women in the U.S. from 2019 to 2020, resulting in a 15% increase in CS rates over this time (from 50.0 to 57.3 cases per 100,000 live births in 2019 and 2020, respectively) [4]. More recently, the CDC reported a 52.3% increase in primary and secondary syphilis rates among women of reproductive age from 2020 to 2021 and a 17.2% increase between 2021 and 2022 [5,13]. Our data support these findings and show that, while the majority of these cases occurred outside of the Northeast, NJ syphilis rates generally followed a similar trend to the rest of the country prior to the COVID-19 pandemic. Further, the spike in CS cases at our hospital above national and state rates in 2020 points towards factors specific to our hospital or patient population that is influencing CS transmission. These may be related to a patient population that is predominantly government-insured or uninsured, or other factors that may influence a city population that is low in access to health care resources.

While we cannot be definitive, the temporal association in our study and other recent studies suggests that the COVID-19 pandemic has only amplified the factors involved in this increase [10]. Nationally reported sexually transmitted infection (STI) rates dropped in early 2020 as national COVID-19 mitigation measures were put into place, despite a steady rise in rates of many STIs in 2020 prior to the implementation of these measures [14]. There are many reasons the pandemic could have had this effect, including the lack of access to health care due to clinic closures, reduced availability of public transportation, shortages of testing materials, and staff reassignment from STI programs to COVID-19 contact tracing that prevented recognition of cases, thus artificially lowering the reported rate [14,15]. These factors could have led to late maternal initiation of care, screening, and treatment that would have prevented infant transmission. Table 3 presents evidence supporting this theory, as it shows that a large proportion of mothers in our study did not receive timely prenatal care, leading to inadequate screening and late initiation of treatment. The effect of the COVID-19 pandemic on CS rates is also represented well by the rate of vertical transmission, which showed a rise in 2020 after dropping to 33.3% in 2019. This suggests that the increase observed was not solely due to increased maternal infection but also to inadequate methods of preventing vertical transmission during the pandemic. In 2021 and beyond, the vertical transmission rate fell back to pre-COVID levels, which further supports the impact of the pandemic on these rates. However, the scientific literature has shown how syphilis and CS rates have been rising since well before the COVID-19 pandemic, and therefore we cannot assume these rates will continue to fall as the pandemic becomes less prominent without additional specific intervention. 

The rate of vertical transmission was interpreted as a measure of the adequacy of our hospital’s screening and treatment protocols. Different states require various levels of screening based on CDC recommendations. New Jersey state law currently mandates serologic screening at the first prenatal visit, in the last trimester, or at delivery for all pregnant women [12]. Standard evaluation involves initial nontreponemal testing using a RPR or VDRL test, with confirmatory treponemal testing for positive screens [16]. A penicillin-based regimen appropriate for the stage of disease is recommended to be initiated >30 days prior to delivery [8]. The diagnosis of CS in an infant is determined by quantitative nontreponemal serologic testing of infant serum and a thorough physical examination [17]. Based on our data, these methods prevent vertical transmission about 50% of the time. The greatest areas of improvement identified in this study included inadequate prenatal care, late maternal initiation of treatment, and inadequate infant follow-up. This corroborates a meta-analysis from Brazil showing prenatal care and adequate maternal treatment as having the highest impact on CS development [18]. Hence, improved monitoring to reduce the risk of vertical transmission as well as reducing the infectious syphilis burden in the general population would go a long way in reducing this burden.

Various social determinants of health are known to impact syphilis transmission. Numerous studies have demonstrated that women of color are more likely to give birth to infants with CS compared to their white counterparts [6,8]. This relationship must be understood in the context of systemic racism, in which women of color may not have the same access to prenatal care as women of other races, leading to poorer detection rates and later initiation of treatment. Aside from race, studies have also noted increased CS incidence among mothers who reported active substance use during pregnancy, recent incarceration, and homelessness [6]. The present study confirms that maternal substance use and homelessness can affect the risk of developing CS. Interestingly, there was a larger proportion of employed women in the CS diagnosis group compared to the unemployed group or those who had unknown employment status. This association has not been reported in the previous literature, and the mechanism may be due to better access to healthcare for the employed patients. 

There are some limitations to the present study. First, our definition of a positive CS case included both proven and possible cases. This was carried out because even unconfirmed cases require proper treatment and follow-up, as defined in the AAP Red Book, to prevent the later development of symptoms. We aimed to evaluate the treatment and screening protocols at our hospital, which required analyzing these possible cases to ensure they were properly followed. However, this could have falsely elevated the case numbers at our hospital. Despite this, the same definition was used throughout the data collection, so the rise in cases we observed was consistent within our dataset. Also, we were limited by our EMR in determining the adequacy of follow-up. If infants followed-up with physicians outside of our medical system, they would be coded as having inadequate follow-up per our methods. This could have falsely elevated the rate of inadequate follow-up. Many charts were also missing data for different variables. This was accounted for by excluding individual charts from the analysis of variables for which data were unavailable. Additionally, per year rates may not have captured changes in case numbers at different time points during the pandemic. Future studies could benefit from analyzing rates on a month-by-month basis after the start of the pandemic, as this could give a better understanding of how rates changed during nationwide lockdowns versus as the lockdowns were lifted. Future studies could also examine patient opinions on how to better engage them in earlier prenatal care with the goal of identifying potential cases earlier and improving rates of appropriate treatment.

## 5. Conclusions

As syphilis rates continue to rise both nationally and internationally, it is crucial for all physicians to be aware of the incidence in their communities and how to best detect and treat the disease, particularly in pregnant patients. This is particularly important in communities at higher risk due to various social factors. Efforts to mitigate the rise in CS should focus on improving access to prenatal care and initiating maternal treatment earlier in pregnancy.

## Figures and Tables

**Table 1:** Maternal Demographics and Social Determinants of Health.

	2016 (n/N; *%*)	2017 (n/N; *%*)	2018 (n/N; *%*)	2019 (n/N; *%*)	2020 (n/N; *%*)	2021 (n/N; *%*)	2022 (n/N; *%*)	Study Total (n/N; *%*)
Mean Age at Delivery	39	27 (range 22–32)	27 (range: 21–29)	20.7 (range: 20–21)	29.7 (range: 23–37)	26.8 (range: 16–41)	26.1 (range: 21–34)	27.3 (range: 16–41)
Race								
White	1/1; 100%	1/2; 50%	1/5; 20%	0/3; 0%	6/15; 40%	3/21; 14.2%	3/14; 21.4%	15/61; 24.6%
Black	0/1; 0%	1/2; 50%	1/5; 20%	1/3; 33.3%	7/15; 46.7%	13/21; 61.9%	5/14; 35.7%	28/61; 45.9%
Hispanic	0/1; 0%	0/2; 0%	1/5; 20%	2/3; 66.7%	1/15; 6.7%	3/21; 14.2%	4/14; 28.6%	11/61; 18.0%
Other	0/1; 0%	0/2; 0%	2/5; 40%	0/3; 0%	1/15; 6.7%	2/21; 9.5%	2/14; 14.3%	7/61; 11.5%
Co-Infection								
HIV	0/1; 0%	0/2; 0%	0/5; 0%	0/3; 0%	1/14; 7.1%	1/20; 5.0%	0/14; 0%	2/59; 3.4%
HepC	1/1, 100%	0/2; 0%	1/5; 20%	0/3; 0%	4/15; 26.7%	5/21; 23.8%	2/14; 14.3%	13/61; 21.3%
Insurance Type								
None	1/1; 100%	0/2; 0%	1/4; 25%	0/3; 0%	0/13; 0%	1/18; 5.6%	0/14; 0%	3/55; 5.5%
Medicare/ Medicaid	0/1; 0%	2/2; 100%	3/4; 75%	2/3; 66.7%	13/13; 100%	17/18; 94.4%	14/14; 100%	51/55; 92.7%
Private	0/1; 0%	0/2; 0%	0/4; 0%	1/3; 33.3%	0/13; 0%	0/18; 0%	0/14; 0%	1/55; 1.8%
Opioid Use at Delivery *	0/1; 0%	0/2; 0%	1/4; 25%	0/2; 0%	6/15; 40%	6/21; 28.6%	4/14; 28.6%	17/59 28.8%
History of Homelessness	0/1; 0%	0/2; 0%	2/4; 50%	0/3; 0%	6/14; 42.9%	6/16; 37.5%	6/13; 46.2%	20/53; 37.7%
History of Incarceration	1/1; 100%	0/2; 0%	1/4; 25%	0/3; 0%	3/13; 23.1%	3/16; 18.8%	6/13; 46.2%	14/52; 26.9%

* Does not include methadone or buprenorphine that the mother received through treatment; N—number of charts with available data included for analysis. Data in this table refer to the 61 maternal cases of syphilis included in the final analysis. HIV: human immunodeficiency virus; HepC: hepatitis C.

**Table 2:** Infant birth characteristics.

	n	N	%
Perinatal Complications *	25	54	46.3
Stillbirth	4	61	6.6
Small for Gestational Age	23	49	46.9
Birth Defects	6	56	10.7

* Include intrauterine growth restriction, preterm labor, premature rupture of membranes, placental abnormalities. N—number of charts included for analysis. Data in this table refers to the 61 infants evaluated in the final analysis.

**Table 3:** Assessment of screening and treatment success.

	Maternal		Infant	
	n/N	%	n/N	%
Appropriate Prenatal Care	18/52	34.6	-	-
Appropriate Screening	32/51	62.7	-	-
Appropriate Treatment	39/48	81.3	40/41	97.6
Treatment > 30 days prior to birth	25/57	43.9	-	-
Appropriate Outpatient Follow-Up	-	-	23/51	45.1

Appropriate prenatal care = ≥1 visit per trimester, appropriate screening = RPR or VDRL testing at 28 weeks’ gestation + in third trimester or at delivery, appropriate treatment = penicillin-based regimen based on disease stage, Appropriate infant outpatient follow-up = visits at 2, 4, 6, and 12 months, with RPR/VDRL every 2–3 months until it became non-reactive.

**Table 4:** Social determinants of health.

	No CS Diagnosis	CS Diagnosis	*p*
	n/N (%)	n/N (%)	
Race			
White	6/27 (22.2)	7/28 (25.0)	0.36
Black	12/27 (44.4)	16/28 (57.1)	
Hispanic	8/27 (29.6)	3/28 (10.7)	
Other	1/27 (3.7)	2/28 (7.1)	
Camden Zip Code	16/27 (59.3)	12/31 (38.7)	0.12
Work Status			
Unemployed	21/27 (77.8)	13/30 (43.3)	0.03
Employed	3/27 (11.1)	8/30 (26.7)	
Unknown	3/27 (11.1)	9/30 (30.0)	
Marital Status			
Single	25/27 (92.6)	21/24 (87.5)	0.78
Married	1/27 (3.7)	2/24 (8.3)	
Other	1/27 (3.7)	1/24 (4.2)	
Concomitant HIV	1/27 (3.7)	1/29 (3.4)	1.0
Concomitant HepC	3/27 (11.1)	9/31 (29.0)	0.09
Alcohol Use *	4/27 (14.8)	3/23 (13.0)	1.0
Smoking *	11/27 (40.7)	15/26 (57.7)	0.22
Past Opioid Use	5/27 (18.5)	12/26 (46.2)	0.03
Current Opioid Use	5/27 (18.5)	10/29 (34.5)	0.18
Other Current Drug Use	11/27 (40.7)	11/27 (40.7)	1.0
Homelessness	6/27 (22.2)	12/23 (52.2)	0.03
Incarceration History	7/27 (25.9)	7/22 (31.8)	0.66

* Alcohol, smoking and other social factors that were present during the current pregnancy. CS: congenital syphilis; HIV: human immunodeficiency virus; HepC: hepatitis C. N—number of charts included for analysis. Not all patients had all data available for analysis.

## Data Availability

The raw data supporting the conclusions of this article will be made available by the authors on request for privacy reasons.
